# ASSESSMENT OF REPRODUCIBILITY OF SANDERS CLASSIFICATION FOR CALCANEAL FRACTURES

**DOI:** 10.1590/1413-785220162402154682

**Published:** 2016

**Authors:** Lucas Gonzaga Piovesana, Hériston Cristovam Lopes, Daniel Moreira Pacca, André Felipe Ninomiya, Mauro César Mattos e Dinato, Rodrigo Gonçalves Pagnano

**Affiliations:** 1. Universidade Estadual de Campinas (Unicamp), Faculdade de Ciências Médicas, Hospital de Clínicas, Campinas, SP, Brazil; 2. Universidade Estadual de Campinas (Unicamp), Faculdade de Ciências Médicas, Campinas, SP, Brazil

**Keywords:** Wounds and Injuries, Calcaneus, Tomography, Reproducibility of results

## Abstract

**Objective:**

: To assess intra- and interobserver reproducibility of Sanders Classification System of calcaneal fractures among experienced and less experienced observers.

**Methods:**

: Forty-six CT scans of intra-articular calcaneal fractures were reviewed. Four observers, two with ten years of experience in foot and ankle surgery and two third-year residents in Orthopedics and Traumatology classified the fractures on two separate occasions three weeks apart from each other. The intra and inter-observer reliability was analyzed using the Kappa index.

**Results:**

: There was good intraobserver reliability for the two experienced observers and one less experienced observer (Kappa values 0.640, 0.632 and 0.629, respectively). The interobserver reliability was fair between the experienced observers (Kappa = 0.289) and moderate among the less experienced observers (Kappa = 0.527).

**Conclusions:**

: The Sanders Classification System showed good intraobserver reliability, but interobserver reproducibility below the ideal level, both among experienced and less experienced observers. Level of Evidence III, Diagnostic Studies.

## INTRODUCTION

Calcaneus fractures are the most common among tarsal bones and are mostly intra-articular fractures with deviation.^1^ Complex calcaneal intra-articular fractures constitute a therapeutic challenge, despite advances in diagnostic imaging, implants for fixation and surgical techniques.

**Figure 1.**
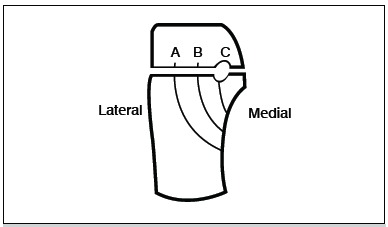
Schematic representation of the talus and calcaneus as observed in the coronal tomography, showing potential sites of fracture in the posterior facet of the calcaneus, used as reference for classification.

The development of computed tomography in the 1980s led to a better understanding of the anatomy of intra-articular fractures of the calcaneus and gave rise to the emergence of various tomographic classifications.^2^ One of the most commonly used classifications is the one by Sanders,^3^ based on the coronal section hindfoot tomography showing the larger lower surface of the posterior facet of the talus. According to this classification, the heel is divided into three columns by two fracture lines A and B. A third line C separates the backbone fragment from the posterior talar facet of the calcaneus, giving rise to four possible articular fragments. (Figure 1) Type 1 is a fracture without deviation, regardless of the number of fragments and type 4 is severely comminuted fractures, with usually four or more parts. Type 2 is essentially a two-part fracture, similar to a longitudinal shear of the tibial plateau, and it is subdivided into types A, B and C, depending on the position of the main fracture line. Type 3 is a fracture in three parts with a central depression similar to a shear-sinking fracture of the tibial plateau, and it is also divided into three parts: AB, BC or AC, depending on the combination of two fracture lines.^4^

The Sanders classification, despite its prognostic value confirmed by several studies,^4^^-^ 6 had its reliability questioned due to low intra- and interobserver reproducibility.^7^^-^ 9 However, there are few studies that have evaluated the classification reproducibility according to the training level of observers.^10^

The primary objective of this study was to evaluate the intra- and interobserver reproducibility of the System of Tomographic Classification of Sanders for calcaneal fractures. Secondly, the reproducibility of classification between less experienced and more experienced observers was also compared.

## MATERIALS AND METHODS

**Figure 2.**
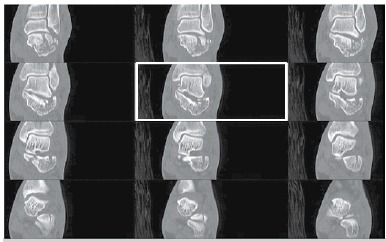
Example of assembly with 12 sequential coronal CT slices of patients with calcaneal fractures present in Power Point files used for classification by observers.

After approval by the Research Ethics Committee of our institution under Nº 45941815.1.0000.5404 CT images of 46 skeletally mature patients of both genders diagnosed with intra-articular fractures of the calcaneus were evaluated. The images were obtained through searching the PACS/PixViewer (Pixeon Medical Sistems S.A., Brazil) database of a tertiary university hospital, which were identified as CT scans of the calcaneus. After obtaining the images, 12 sequential tomographic images of the coronal section that included the entire posterior facet of the calcaneus were selected from each individual. After organizing the images using Power Point software (Figure 2), along with an explanation of how to apply the Sanders classification, the observers independently classified the fractures. The images were selected by an independent researcher who was not involved in the evaluation of CT scans and the identification of survey participants was omitted.

The evaluators were divided into two groups, one consisting of two more experienced senior physicians (specialists in foot and ankle surgery with over ten years of experience) and the other consisting of two less experienced professionals (third-year Orthopedics and Traumatology residents). After 3 weeks of the first assessment the images were presented with a randomly changed test sequence and the same observers classified the fractures once again. The evaluators did not have access to the results of their initial assessments nor to the results of the evaluations of other evaluators involved in the study.

The data obtained in both evaluations were tabulated and statistical analysis of inter- and intraobservers agreement was performed through the Kappa method.^11^ Kappa is a concordance coefficient and its value ranges from 0 to 1. The interpretation of Kappa values, according to Landis and Koch,^12^ agreed with the following values: poor agreement between 0.00 and 0.20; weak between 0.21 and 0.40; moderate between 0.41 and 0.60; substantial or good from 0.61 to 0.81 and almost perfect or excellent agreement between 0.81 and 1.00. The Kappa index was first calculated including eight classification subtypes and then, only among classification types 1 to 4. Statistical analysis was performed using SPSS version 17.0 for Windows.

## RESULTS

In Table 1 we present the classification distribution of the 46 image tests evaluated by the less experienced professionals (observers A and B) and Table 2 shows the rating distribution of the 46 image tests evaluated by the more experienced professionals (observers C and D), including the classification subtypes.

**Table 1.** Frequency distribution of the classification of 46 images performed by less experienced examiners with the subtypes of Sanders classification.

	**Observer A**	**Observador B**						
	**Measurement 1**	**Measurement 2**	**Measurement 1**	**Measurement 2**				
Sanders Classification	N	%	n	%	n	%	n	%
1	3	6.5	5	10.9	3	6.5	4	8.7
2A	7	15.2	13	28.3	18	39.1	3	6.5
2B	15	32.6	7	15.2	7	15.2	20	43.5
2C	1	2.2	0	0.0	0	0.0	0	0.0
3AB	8	17.4	11	23.9	10	21.7	13	28.3
3AC	3	6.5	3	6.5	1	2.2	1	2.2
3BC	3	6.5	3	6.5	2	4.3	2	4.3
4	6	13.1	4	8.7	5	10.9	3	6.5
	46	100.0	46	100.0	46	100.0	46	100.0

**Table 2.** Frequency distribution of the classification of 46 images performed by more experienced examiners with the subtypes of Sanders classification.

	**Observer C**	**Observer D**						
	**Measurement 1**	**Measurement 2**	**Measurement 1**	**Measurement 2**				
Sanders Classification	N	%	n	%	n	%	n	%
1	4	8.7	5	10.9	0	0.0	0	0.0
2A	2	4.3	5	10.9	11	23.9	15	32.6
2B	10	21.7	10	21.7	16	34.8	12	26.1
2C	0	0.0	0	0.0	4	8.7	1	2.2
3AB	10	21.7	10	21.7	4	8.7	7	15.2
3AC	8	17.4	8	17.4	6	13.0	4	8.7
3BC	2	4.3	3	6.5	2	4.3	1	2.2
4	10	21.7	5	10.9	3	6.5	6	13.0
	46	100.0	46	100.0	46	100.0	46	100.0

Examiner A had the same classification in both measurements in 26 images (56.5%) out of 46. The Kappa value for this examiner was 0.634, indicating good agreement. Examiner B had the same classification in both measurements in 13 images (28.3%). The Kappa value for this examiner was 0.325, indicating poor agreement.

Examiner C showed the same classification in both measurements in 25 images (54.3%). The Kappa value for this examiner was 0.640, indicating good agreement. Examiner D had the same classification in both measurements in 27 images (58.7%). The Kappa value for this examiner was 0.632, indicating good agreement on the classifications made.

**Chart 1.**
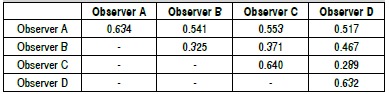
Kappa values for intra- and interobserver of Sanders classification with subtypes.

Chart 1 presents Kappa values ​​for intra- and interobserver, including the classification subtypes. We notice in Chart 1 that the less experienced showed Kappa index 0.541, therefore, moderate agreement. The more experienced observers showed Kappa 0.289, therefore, weak agreement. Examiner A showed moderate agreement with two more experienced examiners (Kappa values 0.553 and 0.517). Examiner B showed weak and moderate agreement with two more experienced examiners (Kappa 0.371 and 0.467, respectively).

Table 3 shows the classification distribution of 46 image exams evaluated by the less experienced (observers A and B) and Table 4 the classification distribution of 46 image exams evaluated by the more experienced (observers C and D), without the classification subtypes.

**Table 3.** Frequency distribution of the classification of 46 images performed by less experienced examiners without subtypes of Sanders classification.

	**Observer A**	**Observer B**						
	**Measurement 1**	**Measurement 2**	**Measurement 1**	**Measurement 2**				
**Sanders Classification**	**n**	**%**	**n**	**%**	**n**	**%**	**n**	**%**
1	3	6.5	5	10.9	3	6.5	4	8.7
2	23	50.0	20	43.4	25	54.4	23	50.0
3	14	30.4	17	37.0	13	28.2	16	34.8
4	6	13.1	4	8.7	5	10.9	3	6.5
	46	100.0	46	100.0	46	100.0	46	100.0

**Table 4.** Frequency distribution of the classification of 46 images performed by more experienced examiners without subtypes of Sanders classification.

	**Observer C**	**Observer D**						
	**Measurement 1**	**Measurement 2**	**Measurement 1**	**Measurement 2**				
**Sanders Classification**	**n**	**%**	**n**	**%**	**n**	**%**	**n**	**%**
1	4	8.7	5	10.9	0	0.0	0	0.0
2	12	26.1	15	32.6	31	67.4	28	60.9
3	20	43.5	21	45.6	12	26.1	12	26.1
4	10	21.7	5	10.9	3	6.5	6	13.0
	46	100.0	46	100.0	46	100.0	46	100.0

Examiner A had the same classification in both measurements in 35 (76.1%) images. The Kappa value for this examiner was 0.667, indicating good agreement. Examiner B had the same classification in both measurements in 27 (58.7%) images. The Kappa value for this examiner was 0.444, indicating moderate agreement.

Examiner C showed the same classification in both measurements in 30 (65.2%) images. The Kappa value for this examiner was 0.628, indicating good agreement. Examiner D had the same classification in both measurements in 36 (78.3%) images. The Kappa value for this examiner was 0.661, indicating good agreement.

**Chart 2.**
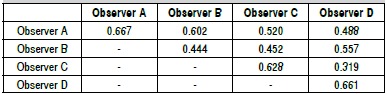
Kappa values for intra- and interobserver of Sanders classification without subtypes.

Chart 2 presents Kappa values for intra- and interobserver without the classification subtypes. We notice in Chart 2 that the less experienced professionals showed Kappa index 0.602, therefore, moderate agreement. The more experienced showed Kappa 0.319, therefore, weak agreement. The two less experienced examiners showed moderate agreement with the two more experienced examiners (Kappa values between 0.452 and 0.557).

## DISCUSSION

Fractures classification systems are an important tool in orthopedics clinical practice, since they may assist in defining treatment and patients' prognosis, and help organizing data for studies conducted in different centers. However, a prerequisite for a classification system to be useful is to be reproducible, both for the same observer in different situations and between different observers.

There are about 15 tomographic classifications available for calcaneal fractures, and Sanders' is among the most frequently used.^5^ Some studies have shown that Sanders classification has a prognostic value,^4^^-^ 6 since it takes into account the location and the number of fracture lines. The more the fragments become medial at the posterior facet of the calcaneus, the more difficult they become regarding surgical access and to perform the reduction.^4^ However, this classification is of little use in making decisions about treatment.^5^^,^ 8 ^,^ 10 Another criticism to the classification is that it would be poorly reproducible,^7^^-^ 9 which, however, does not seem to depend on the evaluator's experience.^10^ This study assessed intra- and interobserver reproducibility and the effect of the training level in the reliability of Sanders classification.

In our study, the level of agreement measured by Kappa index, including all subsets of the classification, showed similar or slightly higher values compared to published studies that evaluated the reproducibility of Sanders classification,^7^^-^ 9 ^,^ 13 with mostly good agreement intraobservers, but with interobserver agreement between weak and moderate. It was interesting to note that interobserver agreement of the more experienced group was lower than in the less experienced group, but in both groups Kappa values for interobserver agreement were below 0.60. Although there is no absolute value to rank reliability as acceptable or not, an index above 0.60 indicates that the classification method is useful.

When subtypes were omitted from the classification, results remained essentially the same, with good intraobserver agreement for most observers and interobserver agreement between weak and moderate. This finding is supported by other studies showing that considering only classification groups 1-4 there is only a small improvement in interobserver agreement.^7^^,^ 8

Among the hypotheses for the good intraobserver agreement and low interobserver agreement is that classification is easily understandable, but its interpretation varies according to the observer, whatever the level of experience. Examiners noticed a common difficulty in choosing between coronal cuts, what would be the one with the widest portion of the posterior facet. Thus, two similar cuts could generate different classifications, revealing a limitation of this classification method. This would explain the low interobserver agreement.

One way proposed to increase the classifications' reliability would be to associate 3D reconstruction images with the analysis of fracture patterns. This feature was not used in this study, since previous studies have found an improvement in inter- and intraobserver reliability for the classification of calcaneal fractures including tridimensional reconstruction images.^14^^,^ 15

Among the limitations of this study is the low number of observers which affects the statistical relevance of the data. However, the methodology was consistent with previous studies which used a similar number of examiners.^7^^-^ 9

Based on the data obtained in our study we observed that, despite the good understanding about Sanders' classification system by observers with different training levels and despite its frequent use, variability in interpretation can make it poorly reproducible.

## CONCLUSIONS

Sanders' Tomographic Classification showed good intraobserver agreement and interobserver reproducibility below ideal, both among more experienced and less experienced observers.
